# Low HDL-Cholesterol Concentrations in Lung Transplant Candidates are Strongly Associated With One-Year Mortality After Lung Transplantation

**DOI:** 10.3389/ti.2023.10841

**Published:** 2023-01-16

**Authors:** Sébastien Tanaka, Christian De Tymowski, Alexy Tran-Dinh, Olivier Meilhac, Brice Lortat-Jacob, Nathalie Zappella, Sylvain Jean-Baptiste, Tiphaine Robert, Tiphaine Goletto, Cendrine Godet, Yves Castier, Hervé Mal, Pierre Mordant, Enora Atchade, Jonathan Messika, Philippe Montravers, the Bichat Lung Transplant Group, Agnès Abadie

**Affiliations:** ^1^ Department of Anesthesiology and Critical Care Medicine, Assistance Publique—Hôpitaux de Paris (AP-HP), Bichat-Claude Bernard Hospital, Paris, France; ^2^ French Institute of Health and Medical Research (INSERM), U1188 Diabetes Atherothrombosis Réunion Indian Ocean (DéTROI), CYROI Platform, Réunion Island University, Saint-Denis de La Réunion, France; ^3^ French Institute of Health and Medical Research (INSERM) U1149, Center for Research on Inflammation, Paris, France; ^4^ UFR Paris Nord, Université Paris Cité, Paris, France; ^5^ Laboratory for Vascular Translational Science, French Institute of Health and Medical Research (INSERM) U1148, Paris, France; ^6^ Reunion Island University-Affiliated Hospital, Saint-Denis, France; ^7^ Department of Biochemistry, Assistance Publique—Hôpitaux de Paris (AP-HP), Bichat-Claude Bernard Hospital, Paris, France; ^8^ Department of Pneumology and Lung Transplantation, Assistance Publique—Hôpitaux de Paris (AP-HP), Bichat-Claude Bernard Hospital, Paris, France; ^9^ PHERE, Physiopathology and Epidemiology of Respiratory Diseases, French Institute of Health and Medical Research (INSERM) U1152, Paris, France; ^10^ Department of Vascular and Thoracic Surgery, Assistance Publique—Hôpitaux de Paris (AP-HP), Bichat-Claude Bernard Hospital, Paris, France; ^11^ Paris Transplant Group, Paris, France

**Keywords:** mortality, lung transplantation, outcome, HDL-cholesterol, lipoprotein

## Abstract

High-density lipoproteins (HDLs), whose main role is the reverse transport of cholesterol, also have pleiotropic anti-inflammatory, antioxidant, anti-apoptotic and anti-infectious properties. During sepsis, HDL cholesterol (HDL-C) concentration is low, HDL particle functionality is altered, and these modifications are correlated with poor outcomes. Based on the protective effects of HDL, we hypothesized that HDL-C levels could be associated with lung transplantation (LT) outcome. We thus looked for an association between basal HDL-C concentration and one-year mortality after LT. In this single-center prospective study including consecutive LTs from 2015 to 2020, 215 patients were included, essentially pulmonary fibrosis (47%) and chronic obstructive pulmonary disease (COPD) (38%) patients. Mortality rate at one-year was 23%. Basal HDL-C concentration stratified nonsurvivors to survivors at one-year (HDL-C = 1.26 [1.12–1.62] mmol/L vs. HDL-C = 1.55 [1.22–1.97] mmol/L, *p* = 0.006). Multivariate analysis confirmed that HDL-C concentration during the pretransplant assessment period was the only variable inversely associated with mortality. Moreover, mortality at one-year in patients with HDL-C concentrations ≤1.45 mmol/L was significantly higher (log-rank test, *p* = 0.00085). In conclusion, low basal HDL-C concentrations in candidates for LT are strongly associated with mortality after LT. To better understand this association, further studies in this field are essential and, in particular, a better characterization of HDL particles seems necessary.

## Graphical Abstract

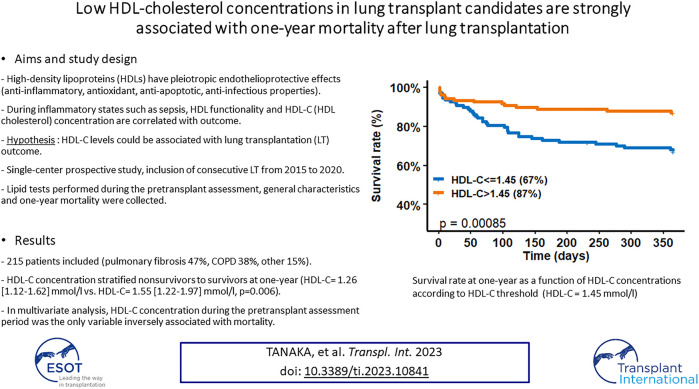

## Introduction

High-density lipoproteins (HDLs) belong to a family of nanoparticles whose main role is the reverse transport of cholesterol from tissues back to the liver (1). A high concentration of HDL-cholesterol (HDL-C) is negatively correlated with the occurrence of acute cardiovascular events such as ischemic stroke or acute coronary syndrome, conferring to this important scavenger role a strong endothelioprotective function (2, 3). Moreover, HDLs have pleiotropic properties that could play a protective role during acute inflammatory states, such as anti-inflammatory, antioxidant, anti-apoptotic and anti-infectious effects (4–7). For example, during sepsis, it has been shown that the HDL-C concentration is low, but HDL particle functionality is also altered, which could potentially impair endothelioprotective function (8–12). During sepsis, these quantitative and qualitative shifts in HDL are highly associated with morbidity and mortality (7–13).

In contrast with atherosclerosis or sepsis, the problematic lipoprotein and lipid levels in the specific case of lung transplantation (LT) have been less studied. Cottini et al. have shown that a low concentration of HDL-C was associated with more primary graft dysfunction (PGD) after LT (14). Moreover, in a retrospective study involving 172 consecutive LT, the same team demonstrated that the total cholesterol (TC)/HDL-C ratio was associated with mortality after LT (15).

The goal of the present study was to assess the lipid profile, particularly the HDL-C levels, in lung transplant candidates in our LT center and to determine a potential relationship with mortality after LT.

## Materials and Methods

### Study Population

All consecutive patients who underwent LT at Bichat-Claude Bernard Hospital, Paris, from January 2015 to December 2020 were prospectively included in this observational, single-center analysis. As lipoprotein concentrations may be affected by liver dysfunction, patients scheduled for liver-lung transplantation were excluded from this study.

According to French law, the patient’s absence of refusal was obtained before inclusion in the study. The Paris-North-Hospitals Institutional Review Board (Paris Diderot University, APHP, IRB No. 0006477) reviewed and approved the study.

### Objectives

The main objective of this study was to investigate any potential association between the basal value of HDL-C and 1-year mortality. The secondary objectives were to assess the association between the basal value of HDL-C and 1-year mortality among patients with chronic obstructive pulmonary disease (COPD) and fibrosis, the two specific subgroups of our population.

### Perioperative Management

The selection of lung transplant candidates (16) and perioperative care (17–20) was standardized for all patients according to current practices. During the intraoperative period, haemodynamic status was monitored using invasive arterial blood pressure, central venous and Swan Ganz catheters, and trans-oesophageal echocardiography. A venoarterial ECMO was implemented in cases of severe pulmonary arterial hypertension, SaO_2_ <85%, SvO_2_ <60%, cardiac output<1.5 L/min/m2 when clamping the pulmonary artery, if the patient did not tolerate single-lung ventilation (hypoxemia or hypercapnia), or in case of respiratory failure after transplantation of the first lung. After the surgical procedure, all patients were managed in a single intensive care unit (ICU). Care in ICU are performed according to international recommendations (21).

### Data Collection

Demographic characteristics during the pretransplant assessment period were prospectively recorded. Mortality at 1 year was also prospectively collected. Occurrence of primary graft dysfunction (PGD), duration of vasopressor agent administration, need of per or postoperative ECMO support, acute kidney injury (AKI), occurrence of digestive complications such as acute mesenteric ischemia (AMI), duration of mechanical ventilation or length of stay in ICU were also collected. Simplified Acute Physiology Score II (SAPS-II) and sepsis-related organ failure assessment (SOFA) score were registered.

Lipid tests were performed during the pretransplant assessment period in the Biochemistry Laboratory of Bichat Claude-Bernard Hospital. This lipid assessment was performed in stable patients without any acute infectious episode. Total cholesterol (TC), HDL-C, LDL-C and triglyceride concentrations were determined by routine enzymatic assays (CHOL, HDLC, LDLC and TRIG methods, Dimension VISTA System, Siemens Healthineers). The reference values for these assays were HDL-C:  >1.40 mmol/L; total cholesterol (TC): 4.40 < N < 5.20 mmol/L and triglycerides: 0.50 < N < 1.7 mmol/L. According to the European Society of Cardiology 2016 recommendations, LDL-C concentration targets have been established depending on vascular risk factors (22). All measurement methods were carried out in accordance with the guidelines.

### Statistical Analysis

Continuous variables are expressed as medians with interquartile ranges (IQRs) and were compared with the Mann–Whitney U test. Categorical variables are expressed as counts and percentages and were compared with Fisher’s exact test or the chi-square test, as appropriate. For 1-year mortality discrimination, receiver operating characteristic curve (ROC) analysis was performed, and the area under the curve (AUC) was calculated. The Youden index was used to determine the best threshold value of HDL-C.

Time-to-event analyses were estimated with Kaplan–Meier analyses. Multivariate associations were computed with binary logistic regression models; variables with nominal 2-tailed *p* values less than 0.2 were entered into the multivariate model, except for variables with obvious collinearity. The final models were selected using backward stepwise regression based on the Akaike information criterion (AIC). All statistical analyses were performed using R statistical software (https://www.r-project.org/). A *p*-value <0.05 was considered statistically significant.

## Results

### Whole Population

#### General Characteristics

Between January 2015 and December 2020, 269 patients underwent LT in our institution. Three patients planned for liver-lung transplantation were excluded from the analysis. Fifty-one patients were excluded from the analysis because their lipid profile was not determined or was incomplete. Overall, 215 patients were finally included in this study. A total of 149 patients (69%) underwent double LT. The patient’s general characteristics during the pretransplant assessment period are presented in Table 1. In this cohort, 10 (4.7%) patients had chronic coronary disease and all these patients underwent a percutaneous coronary intervention. No patient required coronary artery bypass graft (CABG). Moreover, only 10 (4.7%) patients had significant aortic and peripheral vascular calcifications. There was no difference in general characteristics between alive and deceased patients at 1 year.

**TABLE 1:** General characteristics, underlying disease and lipid profile of the overall population stratified by mortality at 1 year.

	Overall population (*n* = 215)	Alive at 1 year (*n* = 166)	Deceased at 1 year (*n* = 49)	p
General characteristics				
Age, years, median [IQR]	57 [51–62]	57 [51–62]	57 [47–62]	0.638
Male sex, *n* (%)	136 (63)	100 (60)	36 (73)	0.091
BMI (kg/m^2^), median [IQR]	24 [20–27]	24 [20–27]	25 [21–28]	0.177
Diabetes mellitus, n (%)	19 (8.8)	12 (7.2)	7 (14)	0.151
Chronic coronary disease n (%)	10 (4.7)	8 (4.8)	2 (4.1)	>0.999
Statin use, n (%)	12 (5.6)	7 (4.2)	5 (10)	0.150
Aortic and peripheral vascular calcifications, n (%)	10 (4.7)	9 (5.4)	1 (2.0)	0.461
Mean pulmonary artery pressure (mmHg), median [IQR]	25 [20–30]	25 [20–30]	26 [20–34]	0.192
Underlying disease leading to LT				
COPD, n (%)	82 (38)	66 (40)	16 (33)	0.368
Pulmonary fibrosis, n (%)	100 (47)	76 (46)	24 (49)	0.693
Other pathologies, n (%)	34 (16)	24 (14)	10 (20)	0.316
Basal lipid profile				
Total cholesterol, mmol/L, median [IQR]	4.97 [4.34–5.70]	5.01 [4.36–5.69]	4.64 [4.33–5.75]	0.427
Triglycerides, mmol/L, median [IQR]	1.18 [0.89–1.65]	1.14 [0.87–1.59]	1.37 [0.97–1.86]	0.057
HDL-C, mmol/L, median [IQR]	1.45 [1.20–1.90]	1.55 [1.22–1.97]	1.26 [1.12–1.62]	0.006
LDL-C, mmol/L, median [IQR]	3 [2.42–3.54]	2.94 [2.40–3.49]	3.16 [2.55–3.55]	0.610

Continuous variables are expressed as the median and interquartile range (IQR) and were compared using the Mann–Whitney U test. Categorical variables are expressed as n (%) and were compared with Fisher’s exact test. BMI, body mass index; COPD, chronic obstructive pulmonary disease; HDL-C, high-density lipoprotein cholesterol; LDL-C, low-density lipoprotein cholesterol; LT, lung transplantation.

#### Blood Lipid Profile

The median delay between the lipid test and LT was 303 [170–552] days. Table 1 presents the lipid profile in the overall population. A comparison between patients alive or deceased at 1 year is also given. In the entire population, the median lipid concentrations were normal, according to the standard values (22). There was no difference between TC, LDL-C and TG concentrations between deceased and alive patients at 1 year. Interestingly, non-survivors had a lower HDL-C concentration than survivors at 1 year (HDL-C = 1.26 [1.12–1.62] mmol/L vs. HDL-C = 1.55 [1.22–1.97] mmol/L, *p* = 0.006).

Moreover, whereas there was no difference in mortality rates at 1 year between patients with and without chronic coronary disease (*p* > 0.999), HDL-C concentrations in patients with chronic coronary disease were lower than in patients without (HDL-C = 1.18 [1.02, 1.30] mmol/L vs. 1.50 [1.21, 1.96] mmol/L, *p* = 0.008). There was no difference in HDL-C concentrations when comparing patients with and without aortic and peripheral vascular calcifications (HDL-C = 1.69 [1.33, 2.30] mmol/L vs. 1.45 [1.20, 1.87] mmol/L, *p* = 0.138).

#### Outcome

The mortality rate at 1 year was 23%. Multivariate analysis with general characteristics during the pretransplant assessment period was performed. These results are expressed in Table 2. A high basal HDL concentration was predictive of good outcomes, suggesting a significant protective effect for 1-year mortality (odds ratio 0.35, 95% CI [0.15, 0.75], *p* = 0.008).

**TABLE 2:** Multivariate analysis of risk factors for mortality at 1 year.

Pretransplant variables	Multivariate analysis of risk factors for mortality at 1 year		
	Odds ratio	95% CI	p
BMI > 25	1.15	0.55–2.37	**0.711**
Male sex	1.14	0.51–2.61	**0.752**
Diabetes mellitus	1.70	0.51–5.21	**0.359**
Statin use	1.42	0.33–5.50	**0.616**
Mean pulmonary artery pressure	1.04	0.99–1.09	**0.092**
Basal TG concentration	1.29	0.78–2.08	**0.303**
Basal HDL-C concentration	0.35	0.15–0.75	**0.008**

BMI, body mass index; HDL-C, high-density lipoprotein cholesterol; TG, triglycerides.

ROC curves were generated to assess the ability of lipid profiles (TC, HDL-C, LDL-C and TG) to discriminate 1-year mortality (Supplementary Figure S1). HDL-C had a higher AUC [0.63 (95% CI 0.54–0.71)]. The best threshold value of HDL-C was 1.45 mmol/L (Youden index, sensitivity = 0.71, specificity = 0.56, positive predictive value (PPV) = 0.32, negative predictive value (NPV) = 0.86).

**FIGURE 1:**
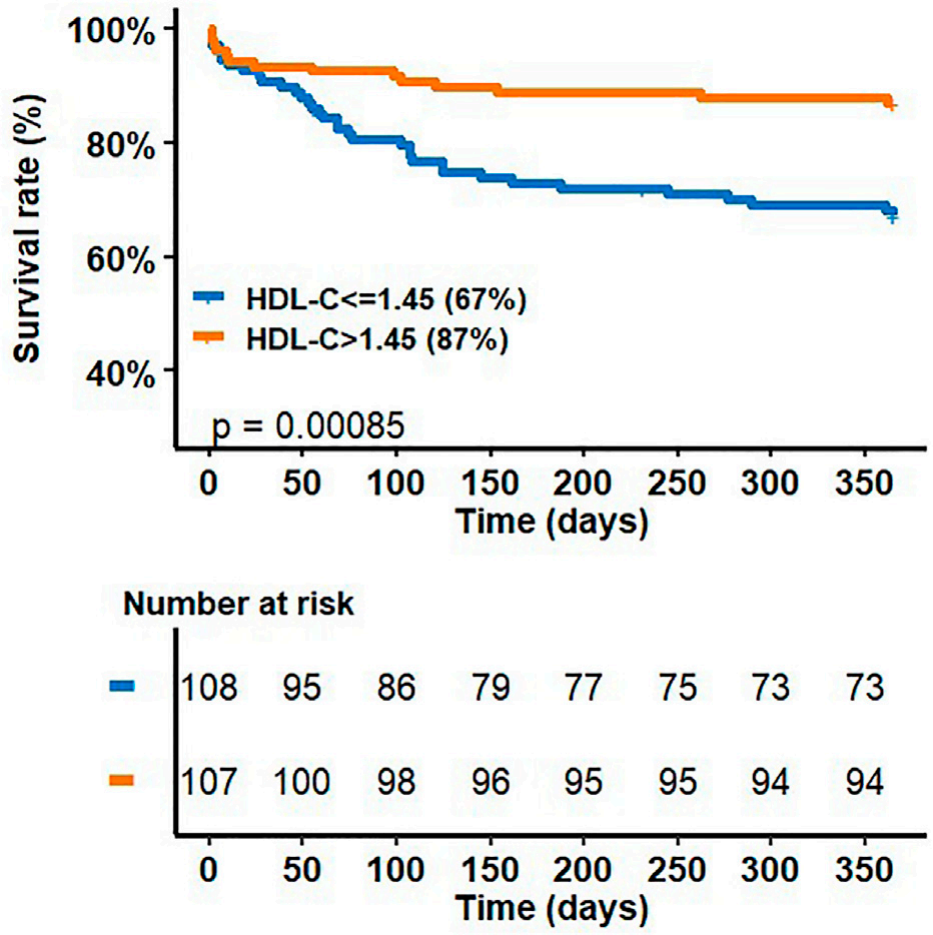
survival rate at 1 year as a function of HDL-C concentrations according to HDL-C threshold (HDL-C = 1.45 mmol/L).

Figure 1 shows the survival rate at 1 year as a function of HDL-C concentrations according to the HDL-C threshold (HDL-C = 1.45 mmol/L). Survival at 1 year of patients with HDL-C concentrations ≤1.45 mmol/L was significantly lower (log-rank test, *p* = 0.00085).

Supplementary Table S1 shows the different values of outcome variables at 1-year mortality.

### Subgroup of COPD Patients

**FIGURE 2:**
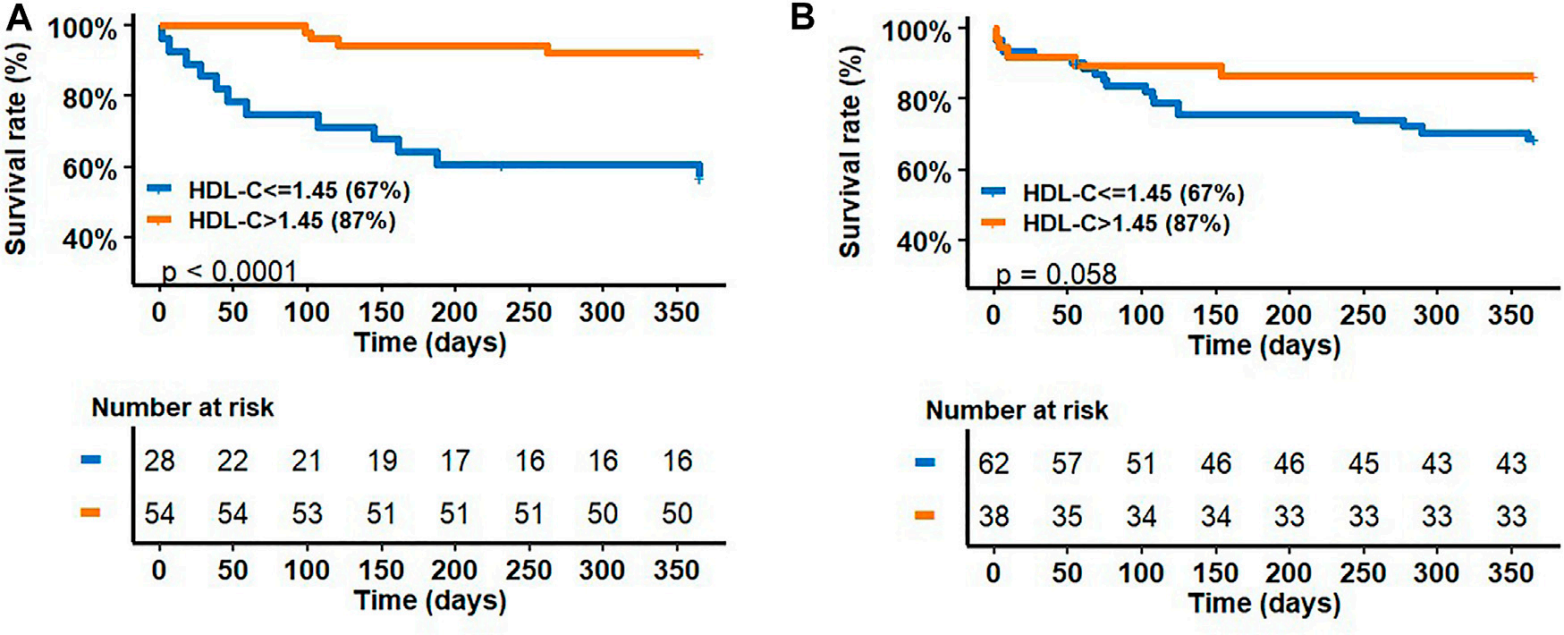
survival rate at 1-year as a function of HDL-C concentrations according to HDL-C threshold (HDL-C = 1.45 mmol/L) in the COPD **(A)** and in fibrosis **(B)** sub-groups.

- Supplementary Table S2 describes the univariate and multivariate analysis of the general characteristics and lipid profile during the pretransplant assessment period and mortality at 1 year in the subgroup of COPD patients. As in the whole population, a high basal HDL-C concentration predicted a good outcome at 1 year in the subgroup of COPD patients (odds ratio 0.13, 95% CI [0.03–0.49], *p* = 0.004).

- ROC curves were constructed to assess the ability of lipid profiles (TC, HDL-C, LDL-C and TG) to discriminate 1-year mortality in the subgroup of COPD patients (Supplementary Figure S2A). HDL-C had a higher AUC [0.76 (95% CI 0.65–0.82)]. The best threshold value of HDL-C was 1.45 mmol/L (Youden index, sensitivity = 0.75, specificity = 0.76, positive predictive value (PPV) = 0.43, negative predictive value (NPV) = 0.93).

- Figure 2A shows the survival rate at 1 year as a function of HDL-C concentrations according to the HDL-C threshold (HDL-C = 1.45 mmol/L). The survival rate at 1 year of patients with HDL-C concentrations ≤1.45 mmol/L was significantly lower (log-rank test, *p* < 0.0001).

### Subgroup of Patients With Fibrosis

- Supplementary Table S3 shows univariate and multivariate analyses of the general characteristics during the pretransplant assessment period and the mortality at 1 year in the subgroup of fibrosis patients. Interestingly, in multivariate analysis, a high TG concentration was significantly associated with 1-year mortality (odds ratio 1.96, 95% CI [1.02–3.85], *p* = 0.044). No relationship between HDL-C concentration and mortality was found.

- ROC curves were generated to assess the ability of lipid profiles (TC, HDL-C, LDL-C and TG) to discriminate 1-year mortality in the subgroup of fibrosis patients (Supplementary Figure S2B). All lipid and lipoprotein values had low AUCs.

- Figure 2B shows the survival rate at 1 year as a function of HDL-C concentrations according to the HDL-C threshold (HDL-C = 1.45 mmol/L). There was no significant difference between survivors and non-survivors patients according to the HDL-C threshold (log-rank test, *p* = 0.058).

## Discussion

The main message of this manuscript is that low basal HDL-C concentration assessed during the pretransplant period is strongly associated with 1-year mortality after LT.

To our knowledge, our study is the first to show this link. Only one previous study has looked at the relationship between the lipid profile and mortality in the context of LT (15). Wenger et al., in a population of 144 LT patients, described a relationship between low basal HDL-C concentration and the occurrence of major cardiovascular events, but did not find any relationship with mortality (15). However, these authors showed that patients who died had a significantly higher TC/HDL-C ratio.

Unlike atherosclerosis or sepsis, where both HDL-C concentration and HDL particle functionality have been extensively studied (8–25), only a few studies have reported HDL-C levels in the context of respiratory disease and LT (26). A retrospective analysis of 126 consecutive individuals evaluated for LT with a diagnosis of COPD showed that HDL-C levels were increased and this was partially attributable to oral steroid use (27). Interestingly, in this study, the HDL-C concentration was not associated with a reduced risk of coronary artery disease. This same team demonstrated that LT in COPD patients led to reductions in the serum levels of HDL-C (28). Moreover, when compared with other populations and, in particular, patients with fibrosis, the HDL-C concentration in COPD patients is very discriminating, which indirectly raises questions about the specific basal metabolism of this population when they are evaluated for LT.

Furthermore, in 69 patients with pulmonary arterial hypertension (PAH), a previous study reported that their HDL-C levels were significantly decreased and were associated with poor clinical outcomes independent of cardiovascular risk factors, insulin resistance and the severity of PAH (29). In patients with idiopathic pulmonary fibrosis (IPF), reduced amounts of apolipoprotein A-I, the major apolipoprotein comprising HDL particles, have been found in bronchoalveolar lavage fluid compared to normal controls (30). Interestingly, the data in our population of fibrosis patients did not highlight an association between basal HDL-C and mortality. The complexity of the fibrosis entity, sometimes occurring in relationship with systemic diseases, may explain, at least partially, the lack of associations. Barochia et al. showed in a study using nuclear magnetic resonance spectroscopy that high levels of small HDL particles (i.e., more functional HDL particles) were negatively correlated with lower mortality or LT (31). Beyond a modification of HDL-C concentration, structural changes of the particles may then influence the outcome.

Since our study was purely exploratory, our observations are only assumptions and it is thus impossible to conclude why we found a strong link between basal HDL-C concentrations and mortality. In light of our results, HDL-C appears to be a marker or an effector in the survival.

Nevertheless, during the per/postoperative periods of LT, acute inflammatory episodes are frequently described, such as systemic inflammatory response syndrome as well as sepsis (19–35). It has been reported that during these states, there is both a decrease in HDL-C and a functional modification of HDL particles with, in particular, a proinflammatory profile (11–42). Importantly, these shifts are associated with poor outcomes. Therefore, if the HDL-C concentration is low under basal conditions and there are both qualitative and quantitative changes during and after LT, the poor prognosis of patients can then be understood.

Our results motivate more powerful clinical studies and experimental studies: If clinical studies confirmed our result, the basal HDL-C concentration could be proposed for discriminating transplant candidates. Moreover, the functionalities of HDL particles before/during/after LT probably deserve additional investigation. Whereas during sepsis, functional and structural changes have been well described (7), no study has yet investigated these functionalities in LT. Studies, in particular -omics analyses, seem necessary to better characterize these LT candidates.

Our study has several limitations:

First, this investigation being purely observational, we have no element that can rationally explain our results. Second, the monocentric nature of our work with only 215 patients is a limitation, which can lead to recruitment bias. Third, the mixture of varied respiratory pathologies (COPD and fibrosis) can also bring about some biases. A focus on the specific COPD candidates could be very informative. Fourth, an analysis of HDL-C concentrations per and postoperatively would have been informative. Fifth, even if 1-year mortality is not a reliable indicator of LT transplant center performance (43), our mortality rate at 1 year (23%) seems to be slightly high and this could induce some bias. The high proportion of fibrosis and high emergency LT (18%) could possibly explain this elevated rate. Finally, whereas there was no difference in mortality rates at 1-year between patients with and without chronic coronary disease, HDL-C concentration in patients with chronic coronary disease were lower than in patients without. Even if this population is very small (*n* = 10), it could induce a bias.

In conclusion, our work showed that a low basal HDL-C concentration in candidates for LT was associated with increased mortality after LT. HDL-C appears to be a marker or an effector in the survival. To better understand this association, additional and more powerful studies are required. A better characterization of HDL particles is also a huge challenge.

## Investigators of the Bichat Lung Transplant Group

Agnès Abadie, Enora Atchade, Mouna Ben-rehouma, Vincent Bunel, Yves Castier, Pierre Cerceau, Diego Ferreira, Gwenn Frere, Lucie Genet, Cendrine Godet, Tiphaine Goletto, Sylvain Jean-Baptiste, Gilles Jebrak, Elie Kantor, Paul Labed, Dan Longrois, Brice Lortat-Jacob, Hervé Mal, Armelle Marceau, Chahine Medraoui, Charlotte Thibaut de Menonville, Jonathan Messika, Alexandre Mignon, Domitille Mouren, Quentin Pellenc, Régis Renard, Arnaud Roussel, Mathilde Salpin, Léa Copelovici, Sandrine Boudinet, Alice Savary, Jean Senemaud, Aurélie Snauwert, Jules Stern, Sebastien Tanaka, Parvine Tashk, Sandrine Tissot, Alexy Tran-Dinh, Sabrina Trigueiros, Christian de Tymowski, Gaëlle Weisenburger, Nathalie Zappella.

## Data Availability

The raw data supporting the conclusion of this article will be made available by the authors, without undue reservation.

## Abbreviations

AIC, akaike information criterion; AUC, area under the curve; COPD, chronic obstructive pulmonary disease; HDL, high-density lipoprotein; HDL-C, HDL cholesterol; ICU, intensive care unit; LDL, low-density lipoprotein; LDL-C, LDL cholesterol; LT, lung transplantation; NPV, negative predictive value; PPV, positive predictive value; ROC, receiver operating characteristic; TC, total cholesterol; TG, triglyceride.
