# Supplementary material for: Low HDL-Cholesterol Concentrations in Lung Transplant Candidates are Strongly Associated With One-Year Mortality After Lung Transplantation
Source: Transpl Int. 2023 Jan 16;36:10841. doi: 10.3389/ti.2023.10841 (PMC9884674; doi:10.3389/ti.2023.10841)
Supplement: Supplementary file 1 [file Table1.DOCX]

Supplemental Table S1 : Outcome variables stratified by mortality at one year.

| **Per/postoperative and outcome variables** | **Overall population**  **(n = 215)** | **Alive**  **at one-year**  **(n = 166)** | **Deceased**  **at one-year**  **(n = 49)** | **p** |
| --- | --- | --- | --- | --- |
| SAPSII score on ICU admission, median [IQR] | 43 [38, 52] | 43 [38, 50] | 48 [43, 70] | <0.001 |
| SOFA on ICU admission, median [IQR] | 7 [6, 9] | 7 [6, 9] | 8 [7, 10] | 0.001 |
| Duration of vasopressor agent administration, days, median [IQR] | 2.0 [1.0, 4.0] | 2.0 [1.0, 3.0] | 3.0 [1.0, 9.0] | 0.002 |
| Need of per and postoperative ECMO support, n (%) | 149 (69) | 106 (64) | 43 (88) | 0.001 |
| Stage III PGD, n (%) | 38 (18) | 25 (15) | 13 (27) | 0.064 |
| Acute kidney injury, | 119 (55) | 82 (49) | 37 (76) | 0.001 |
| Acute kidney injury, KDIGO stage 3, n (%) | 28 (13) | 9 (5.4) | 19 (39) | <0.001 |
| Renal replacement therapy, n (%) | 22 (10) | 6 (3.6) | 16 (33) | <0.001 |
| acute mesenteric ischemia, n (%) | 6 (2.8) | 2 (1.2) | 4 (8.2) | 0.025 |
| Duration of MV, days, median [IQR] | 3 [1, 16] | 3 [1, 10] | 6 [2, 49] | <0.001 |
| ICU length of stay, median [IQR] | 17 [10, 33] | 16 [11, 28] | 20 [8, 55] | 0.522 |

Continuous variables are expressed as median and interquartile range (IQR) and were compared using the Mann-Whitney U test. Categorical variables are expressed as n (%) and were compared with Fisher's exact test. ECMO, extracorporeal membrane oxygenation; ICU, intensive care unit; KDIGO, kidney disease improving global outcomes; MV, mechanical ventilation; PGD, primary graft dysfunction; SAPS-II, Simplified Acute Physiology Score II ; SOFA, sepsis-related organ failure assessment.
