# Supplementary figures and images for: Low HDL-Cholesterol Concentrations in Lung Transplant Candidates are Strongly Associated With One-Year Mortality After Lung Transplantation
Source: Transpl Int. 2023 Jan 16;36:10841. doi: 10.3389/ti.2023.10841 (PMC9884674; doi:10.3389/ti.2023.10841)

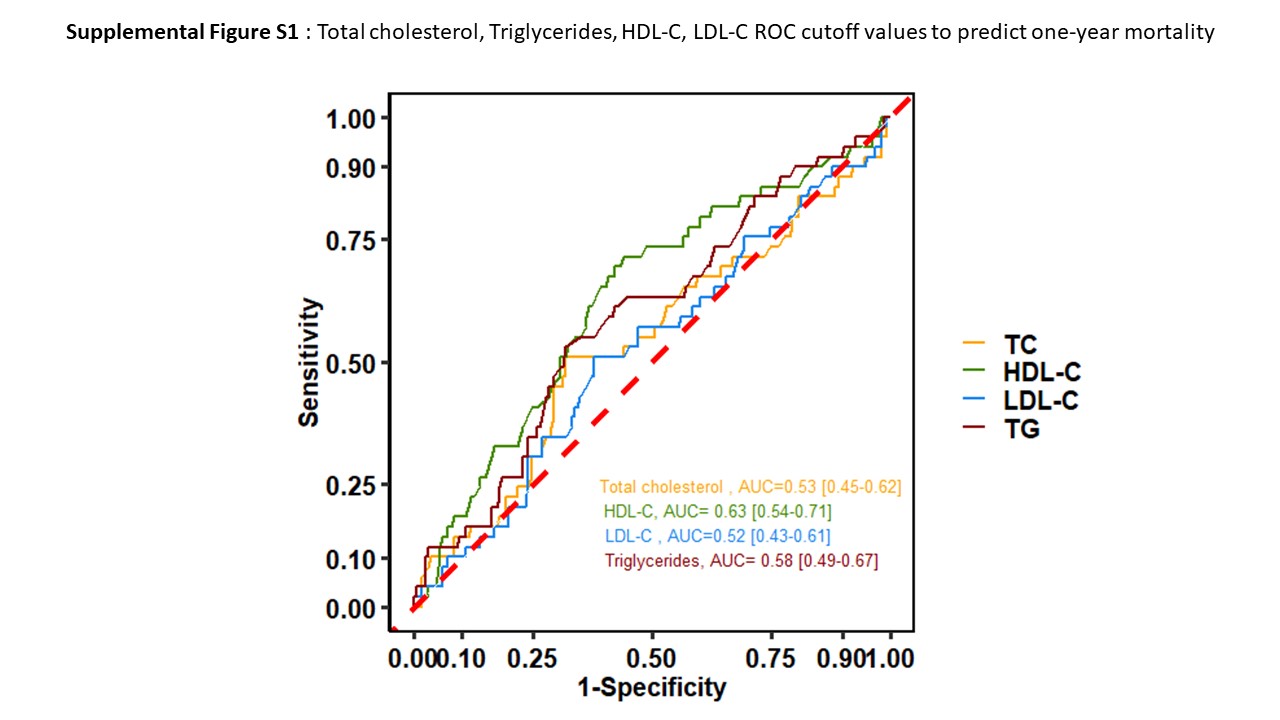

Supplement: Supplementary file 2 [file Image1.JPEG]

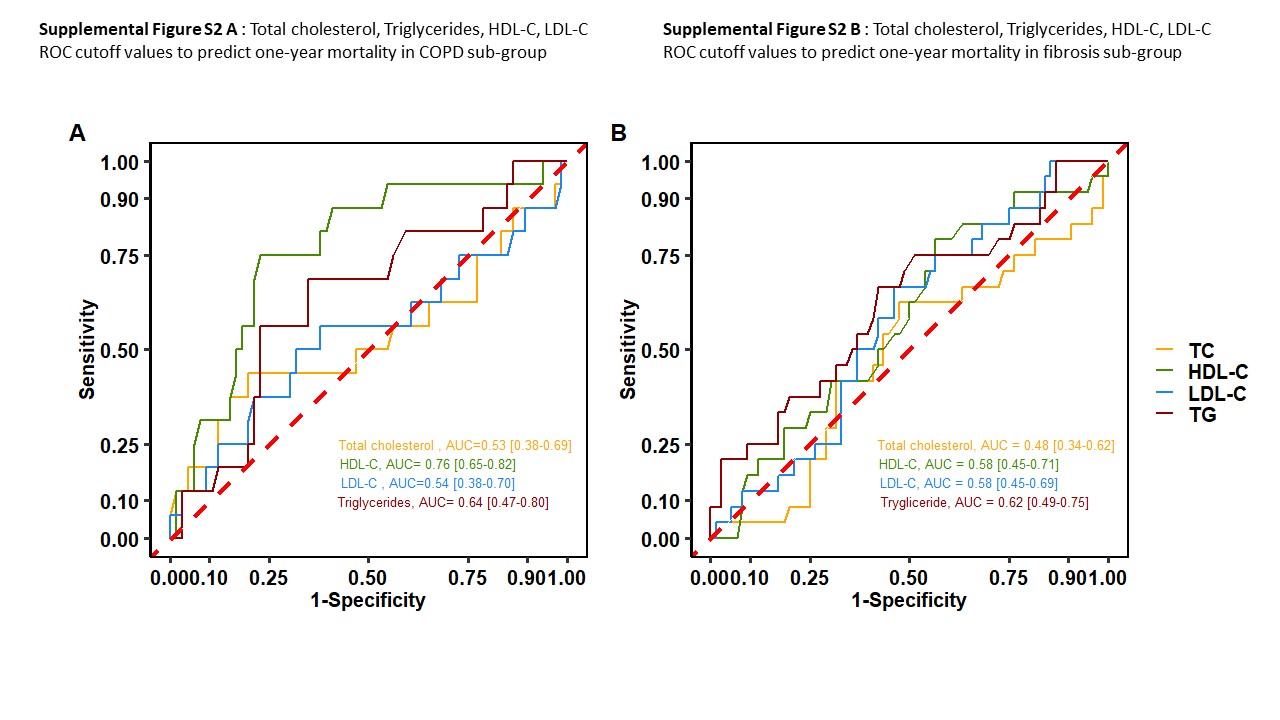

Supplement: Supplementary file 3 [file Image2.JPEG]
