# Supplementary material for: Low HDL-Cholesterol Concentrations in Lung Transplant Candidates are Strongly Associated With One-Year Mortality After Lung Transplantation
Source: Transpl Int. 2023 Jan 16;36:10841. doi: 10.3389/ti.2023.10841 (PMC9884674; doi:10.3389/ti.2023.10841)
Supplement: Supplementary file 4 [file Table2.DOCX]

Supplemental Table S2: Relationship between general characteristics and mortality at one year in the subgroup of COPD patients

| **Variables** | **Univariate analysis** | | | | **Multivariate analysis** | | |
| --- | --- | --- | --- | --- | --- | --- | --- |
|  | **Overall population**  **(n = 82)** | **Alive at one year**  **(n = 66)** | **Deceased at one year**  **(n = 16)** | ***p* value** | **Odd-ratio** | **95% CI** | ***p* value** |
| Age, years, median [IQR] | 58 [54-62] | 58 [54-62] | 59 [53-62] | 0.865 |  |  |  |
| Male sex, *n* (%) | 47 (57) | 35 (53) | 12 (75) | 0.111 | 1.18 | [0.29-5.21] | 0.816 |
| BMI (kg/m^2^), median [IQR] | 23 [20-27] | 22 [19-27] | 24 [21-26] | 0.660 |  |  |  |
| Diabetes mellitus, n (%) | 2 (2.4) | 1 (1.5) | 1 (6.2) | 0.354 |  |  |  |
| Chronic coronary disease, n (%) | 3 (3.7) | 2 (3.0) | 1 (6.2) | 0.483 |  |  |  |
| Statin use, n (%) | 5 (6.1) | 1 (1.5) | 4 (25) | 0.005 | 13.89 | [1.34-464] | 0.059 |
| Mean pulmonary artery pressure (mmHg), median [IQR] | 27 [22-30] | 26 [22-30] | 27 [24-34] | 0.311 |  |  |  |
| Double LT, n (%) | 63 (77) | 49 (74) | 14 (88) | 0.338 |  |  |  |
| Total cholesterol, mmol/l, median [IQR] | 4.96 [4.57-5.79] | 4.96 [4.64-5.74] | 5.02 [4.23-6.07] | 0.712 |  |  |  |
| Triglycerides, mmol/l,  median [IQR] | 1.08 [0.79-1.57] | 1.04 [0.79-1.36] | 1.46 [0.96-1.69] | 0.091 | 0.89 | [0.36-1.99] | 0.786 |
| HDL-C, mmol/l, median [IQR] | 1.73 [1.26, 2.05] | 1.79 [1.46-2.10] | 1.25 [1.15-1.46] | 0.001 | 0.13 | [0.03-0.49] | 0.004 |
| LDL-C, mmol/l, median [IQR] | 2.83 [2.39, 3.49] | 2.81 [2.39-3.38] | 3.08 [2.32 3.85] | 0.631 |  |  |  |

Continuous variables are expressed as the median and interquartile range (IQR) and were compared using the Mann–Whitney U test. Categorical variables are expressed as n (%) and were compared with Fisher's exact test. BMI, body mass index; HDL-C, high-density lipoprotein cholesterol; LDL-C, low-density lipoprotein cholesterol; LT, lung transplantation
