# Supplementary material for: Low HDL-Cholesterol Concentrations in Lung Transplant Candidates are Strongly Associated With One-Year Mortality After Lung Transplantation
Source: Transpl Int. 2023 Jan 16;36:10841. doi: 10.3389/ti.2023.10841 (PMC9884674; doi:10.3389/ti.2023.10841)
Supplement: Supplementary file 5 [file Table3.DOCX]

Supplemental Table S3: relationship between general characteristics and mortality at one year in the sub-group of patients with fibrosis

| **Variables** | **Univariate analysis** | | | | **Multivariate analysis** | | |
| --- | --- | --- | --- | --- | --- | --- | --- |
|  | **Overall population**  **(n = 100)** | **Alive at one-year**  **(n = 76)** | **Deceased at one-year**  **(n = 24)** | ***p* value** | **Odd-ratio** | **95% CI** | ***p* value** |
| Age, years, median [IQR] | 58 [53-63] | 58 [53-63] | 59 [53-63] | 0.843 |  |  |  |
| Male sex, *n* (%) | 73 (73) | 55 (72) | 18 (75) | 0.800 |  |  |  |
| BMI (kg/m^2^), median [IQR] | 25.0 [22.0-28.0] | 24.0 [22.0-28.0] | 27.0 [24.3-29.0] | 0.057 | 2.58 | [0.94-7.67] | 0.073 |
| Diabetes mellitus, n (%) | 13 (13) | 8 (11) | 5 (21) | 0.293 |  |  |  |
| Chronic coronary disease, n (%) | 7 (7.0) | 6 (7.9) | 1 (4.2) | >0.99 |  |  |  |
| Statin use, n (%) | 6 (6.0) | 5 (6.6) | 1 (4.2) | >0.99 |  |  |  |
| Mean pulmonary artery pressure (mmHg), median [IQR] | 6 (6.0) | 5 (6.6) | 1 (4.2) | >0.99 |  |  |  |
| Double LT, n (%) | 55 (55) | 44 (58) | 11 (46) | 0.30 |  |  |  |
| Total cholesterol, mmol/l, median [IQR] | 5.16 [4.38-5.72] | 5.19 [4.35-5.70] | 5.06 [4.45-5.79] | 0.774 |  |  |  |
| Triglycerides, mmol/l,  median [IQR] | 1.36 [1.04-1.80] | 1.28 [1.04-1.71] | 1.48 [1.22-2.04] | 0.076 | 1.96 | [1.02-3.85] | **0.044** |
| HDL-C, mmol/l, median [IQR] | 1.33 [1.14-1.59] | 1.35 [1.15-1.61] | 1.29 [1.07-1.44] | 0.047 | 0.81 | [0.22-2.74] | 0.740 |
| LDL-C, mmol/l, median [IQR] | 3.18 [2.70-3.79] | 3.12 [2.61-3.81] | 3.37 [2.96-3.61] | 0.307 |  |  |  |

Continuous variables are expressed as median and interquartile range (IQR) and were compared using the Mann-Whitney U test. Categorical variables are expressed as n (%) and were compared with Fisher's exact test. BMI, body mass index; HDL-C, high-density lipoprotein cholesterol; LDL-C, low-density lipoprotein cholesterol; LT, lung transplantation
